# Comparison of particle image velocimetry and the underlying agents dynamics in collectively moving self propelled particles

**DOI:** 10.1038/s41598-023-39635-z

**Published:** 2023-08-02

**Authors:** Udoy S. Basak, Sulimon Sattari, Md. Motaleb Hossain, Kazuki Horikawa, Mikito Toda, Tamiki Komatsuzaki

**Affiliations:** 1 Pabna University of Science and Technology, Pabna, 6600 Bangladesh; 2 Research Center of Mathematics for Social Creativity, Research Institute for Electronic Science, Hokkaido University, Kita 20, Nishi 10, Kita-ku, Sapporo, 001-0020 Japan; 3 University of Dhaka, Dhaka, 1000 Bangladesh; 4 Department of Optical Imaging, Advanced Research Promotion Center, Tokushima University, Kuramoto-cho 3-18-15, Tokushima, Tokushima 770-8503 Japan; 5 Faculty Division of Natural Sciences, Research Group of Physics, Nara Women’s University, Kita-Uoya-Nishimachi, Nara, 630-8506 Japan; 6 Graduate School of Information Science, University of Hyogo, 7-1-28 Minatojima-minamimachi, Chuo-ku, Kobe, Hyogo 650-0047 Japan; 7 Research Institute for Electronic Science, Hokkaido University, Kita 20 Nishi 10, Kita-Ku, Sapporo, 001-0020 Japan; 8 Graduate School of Life Science, Transdisciplinary Life Science Course, Hokkaido University, Kita 12, Nishi 6, Kita-ku, Sapporo, 060-0812 Japan; 9 Institute for Chemical Reaction Design and Discovery (WPI-ICReDD), Hokkaido University, Kita 21 Nishi 10, Kita-ku, Sapporo, Hokkaido 001-0021 Japan; 10 Graduate School of Chemical Sciences and Engineering Materials Chemistry and Engineering Course, Hokkaido University, Kita 13, Nishi 8, Kita-ku, Sapporo, 060-0812 Japan; 11 Institute for Open and Transdisciplinary Research Initiatives, Osaka University, Yamadaoka, Suita, Osaka 565-0871 Japan; 12 The Institute of Scientific and Industrial Research, Osaka University, Mihogaoka, 8-1, Osaka, Ibaraki 567-0047 Japan

**Keywords:** Biological techniques, Biophysics, Computational biology and bioinformatics, Physics

## Abstract

Collective migration of cells is a fundamental behavior in biology. For the quantitative understanding of collective cell migration, live-cell imaging techniques have been used using e.g., phase contrast or fluorescence images. Particle tracking velocimetry (PTV) is a common recipe to quantify cell motility with those image data. However, the precise tracking of cells is not always feasible. Particle image velocimetry (PIV) is an alternative to PTV, corresponding to Eulerian picture of fluid dynamics, which derives the average velocity vector of an aggregate of cells. However, the accuracy of PIV in capturing the underlying cell motility and what values of the parameters should be chosen is not necessarily well characterized, especially for cells that do not adhere to a viscous flow. Here, we investigate the accuracy of PIV by generating images of simulated cells by the Vicsek model using trajectory data of agents at different noise levels. It was found, using an alignment score, that the direction of the PIV vectors coincides with the direction of nearby agents with appropriate choices of PIV parameters. PIV is found to accurately measure the underlying motion of individual agents for a wide range of noise level, and its condition is addressed.

## Introduction

Cells perform organized, ‘orchestrated’ movement known as collective cell migration^1,2^. Their migration is crucial during embryo and organ development^3,4^, wound healing^1,5^, cancer growth^6^, and immune defense^7^. The failure of cells to cooperate may result in life-threatening concerns^1,8^. How do cells harmonize with each other during migration, given that individual cells have limited sensory, communication, and decision-making abilities? In some cases, collective behavior is coordinated by leader cells, or cells whose behavior is more influential than others, however, the existence and role of leader cells are still widely unknown for different cell lines^9–12^. Quantitative measurement of cell velocities can shed light on how they coordinate. Recently, improvements in image acquisition have resulted in high-resolution, high-frame rate, images with unprecedented viewing range^13–19^, which provide rich information on collective cell motion.

Particle tracking velocimetry (PTV) is a well-known technique where individual cells are tracked, resulting in a so-called “Lagrangian description” of the velocity dynamics. PTV usually contains two steps—a segmentation step, where the locations of cells and their boundaries are identified^20–22^, and a tracking step, where the location of each cell in one frame is associated with the location of the same cell in the next frame. PTV is onerous when done manually for many cells, and, when automated using supervised and unsupervised techniques, can lead to erroneous results when cell density is high or cells move rapidly compared to the image acquisition rate^23–25^. In segmentation, a cell may be missed, or something which is not a cell can be marked as a cell. In tracking, a cell can be associated with another (incorrect) cell in the next frame. Furthermore, for the supervised methods, a fair amount of training data set is needed, whose labels must be assigned manually^20^.

Alternatively, one can obtain an Eulerian description of the velocity field, where, instead of identifying individual cells, flow properties are expressed as a field. Particle image velocimetry (PIV) is a technique that has been used widely in cell migration analysis^26–30^ to obtain Eulerian velocity field dynamics from sequences of images. In PIV, each image is first divided into interrogation regions (grids), and each grid area in a given frame is associated with the corresponding grid area in the next frame with the highest cross-correlation^31^. Previously, the efficacy of the PIV technique has been assessed to a sequence of images generated by applying a known flow mapping to images of cells, to see how the vectors outputted by PIV can accurately track the deformations in the image by the flow field over time. It was shown that PIV works well in these cases^23^. However, it is yet to be scrutinized the performance of PIV when cells move in an incoherent fashion, or when the system changes from disordered motions to some coherent state. In many cell lines, such as *Dictyostelium discoideum*, cells do not move by any prescribed viscous flow, and instead, travel like stochastic particles with some couplings. In order to verify the effectiveness of PIV in reconstructing the trajectories of cells which do not travel in a viscous flow, one may take the help of simulation models where the actual trajectories are known.

In this paper, we investigate the accuracy of PIV in capturing the motion of particles, not in a prescribed viscous flow, but the particles spontaneously move with their mutual interference under the presence of noise by the Vicsek Model (VM)^32^. Here noise emerges from the particles’ “failure” to accurately follow the motion of their neighbors. Using the trajectory data of particles at different noise levels, we produce images of the agents by plotting filled ovals at each particle location which are oriented following the interaction rules of the VM. The PIV technique is applied to the images alone without having access to the original trajectory data. We then compare the velocities computed by PIV to the velocities known from the trajectories using an alignment score, $A^R$, we introduce in this paper. Here, *R* denotes a given radius of a circle whose center coincides with each PIV position so that all particles dynamics passing through inside the circle are taken into account. The alignment score has revealed that the PIV vectors’ direction matches the direction of the adjacent particles, especially at low noise levels where the system exhibits global coherence. For higher noise cases, the selection of a precise PIV grid size and *R* values have proven to be crucial. It has been seen that the alignment score increases significantly upon incorporating such *R* values such that agents are relatively coherent with each other within that radius. As the noise level gets higher, the agents get more randomized so that the larger the PIV grid size, the more the PIV vectors quickly deviate from the underlying agents’ dynamics. In other words, PIV can still capture the dynamics inherent to the entities of the agents if we use the right PIV grid size in which just a single agent exists on average.

**Figure 1:**
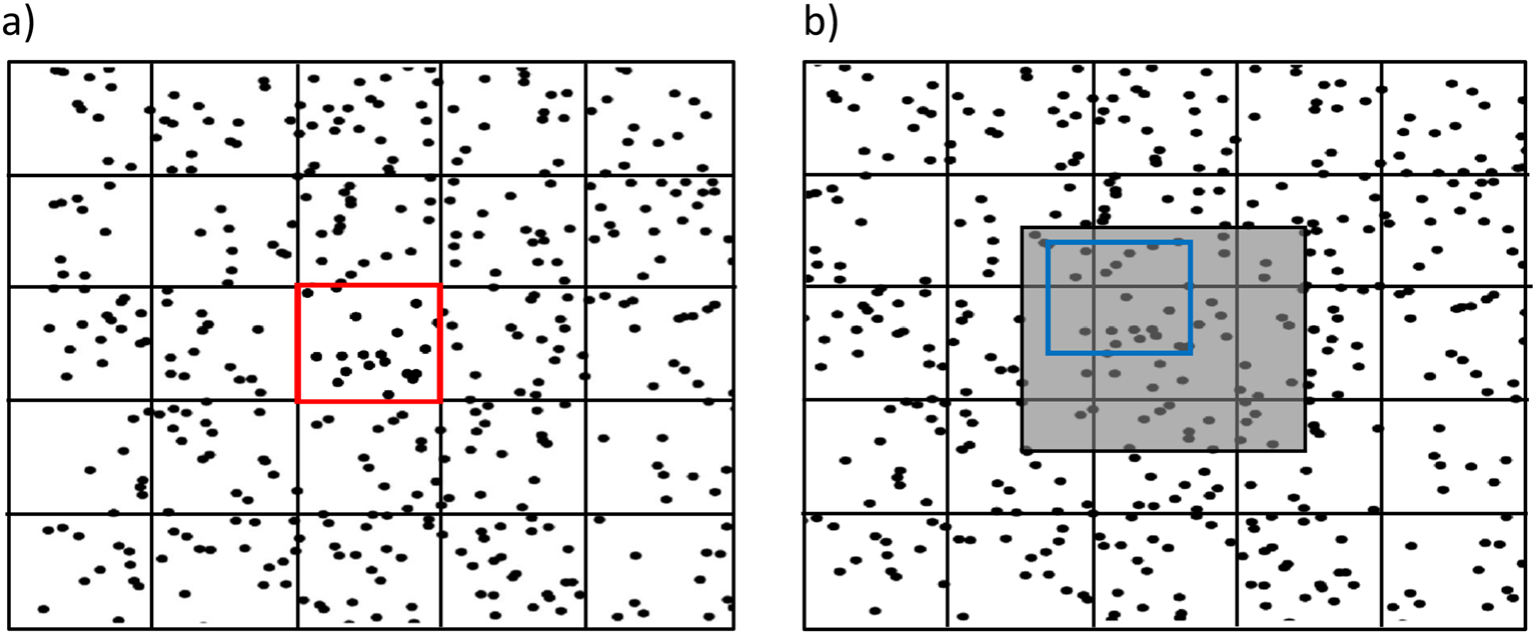
Schematic diagram of moving particles at time (**a**) $t$ and (**b**) $t+1$. Small black boxes in both figures represent a division of the original image into grids, and for each grid, a PIV vector is defined that estimates the mean displacement vector of particles within that grid. The shaded region in (**b**) represents the interrogation zone of the red grid in (**a**) within which the matched area may exist. The size of the interrogation window largely depends on the distance traveled by the particles in the time interval between two successive images. The blue box inside the search zone represents the window whose center is varied within the search zone. The orientation of agents within the red grid and windows is computed to find the best-matched window.

**Figure 2:**
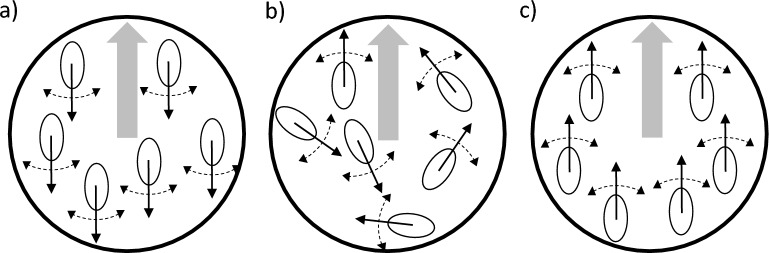
Schematic representation of groups of particles with varying alignment score $A^R$ where the thick-shaded arrow represents a PIV vector defined over a grid, and the ovals with arrows stand for the moving particles along with their directions of movement. (**a**) The direction of the PIV vector is the opposite of the direction in which the particles move. Therefore, $A^R$ will be close to $-1$ where *R* is the radius of the circle. (**b**) Particles move randomly hence $A^R$ will be close to 0 for such cases. (**c**) The direction of the PIV vector is aligned to the direction in which the particles move, hence $A^R$ will be close to unity.

## Averaged normalized velocity and alignment score

We now describe the PIV scheme and also several measures that have been investigated as ways to quantify the efficiency of PIV in scrutinizing collective migration.

### Particle image velocimetry (PIV)

PIV computes Eulerian velocity fields, that is, it measures a flow field as the fluid passes an observation point that is fixed in space. PIV does not use image segmentation, instead, it computes velocity vectors by searching for a zone in a future frame that matches best with a given grid in the current frame. Figure 1 shows two consecutive images of moving particles at time $t$ (Fig. 1a) and $t+1$ (Fig. 1b). At the beginning of the process, each image is divided into grids as shown in Fig. 1, and the center of each grid remains the same in all images. For each grid at time *t*, say the red grid in Fig. 1a, our goal is to find the zone at a subsequent image at time $t+1$ where the image in the grid at time *t* matches best with the image in the zone at time $t+1$. Instead of searching the whole image to find the best match of the red grid, an interrogation zone which is typically a square of size $N_g \times N_g$ pixels is defined in the succeeding image (shaded region in Fig. 1b) where $N$ is chosen to be roughly equal to the largest possible displacement of agents within two successive frames. Then PIV creates a temporary grid (we refer to it as a window) within the search zone at time $(t+1)$ (blue box in Fig. 1b) of the same size as the red grid. We then search the window to match the orientation of particles within the red grid. The location of the window is then varied within the search zone to find the best match. The process of finding the best match for the displacements of grids between two subsequent images is performed by cross-correlation having the following form^23^:

1 $$\begin{aligned} C(k,l)=\frac{\sum _{i=1}^{Z}\sum _{j=1}^{Z} [I^t(i,j)-\mu _{I^t}][I^{t+1}(i+k,j+l)-\mu _{I^{t+1}}]}{\sqrt{\sum _{i=1}^{Z}\sum _{j=1}^{Z} [I^{t}(i,j)-\mu _{I^{t}}]^2}\sqrt{\sum _{i=1}^{Z}\sum _{j=1}^{Z} [I^{t+1}(i+k,j+l)-\mu _{I^{t+1}}]^2}} \end{aligned}$$

where $I^{t}(i,j)$ and $I^{t+1}(i+k,j+l)$ denote the intensity of images at the positions (*i*, *j*) and $(i+k,j+l)$ at time $t$ and $t+1$, respectively. Here *Z* represents the number of pixels along one side of the (square) grid. Here $\mu _{I^{t}}$ represents the average intensity of a grid at time $t$ (red grid, say), and $\mu _{I^{t+1}}$ is the average intensity of a grid located within the interrogation window (e.g., blue grid) at time $t+1$. (*k*, *l*) is the displacement of the (red) grid from its original location. The correlation function *C*(*k*, *l*) defined in Eq. (1) is maximized when the overlapping part between the red grid (Fig. 1a) and its best matched location at two consecutive images is maximum, and the location of this peak in the correlation then defines how far the (red) grid has moved between the two consecutive images. Finally, PIV draws a vector starting from the center of the (red) grid to the center of its best-matched window. The length of the vector represents the distance that the particles within the (red) grid travel during these consecutive time frames. Hence, a PIV vector characterizes the aggregate motion of particles located within a grid, and by studying all the PIV vectors in the system one can surmise the way particles move.

**Figure 3:**
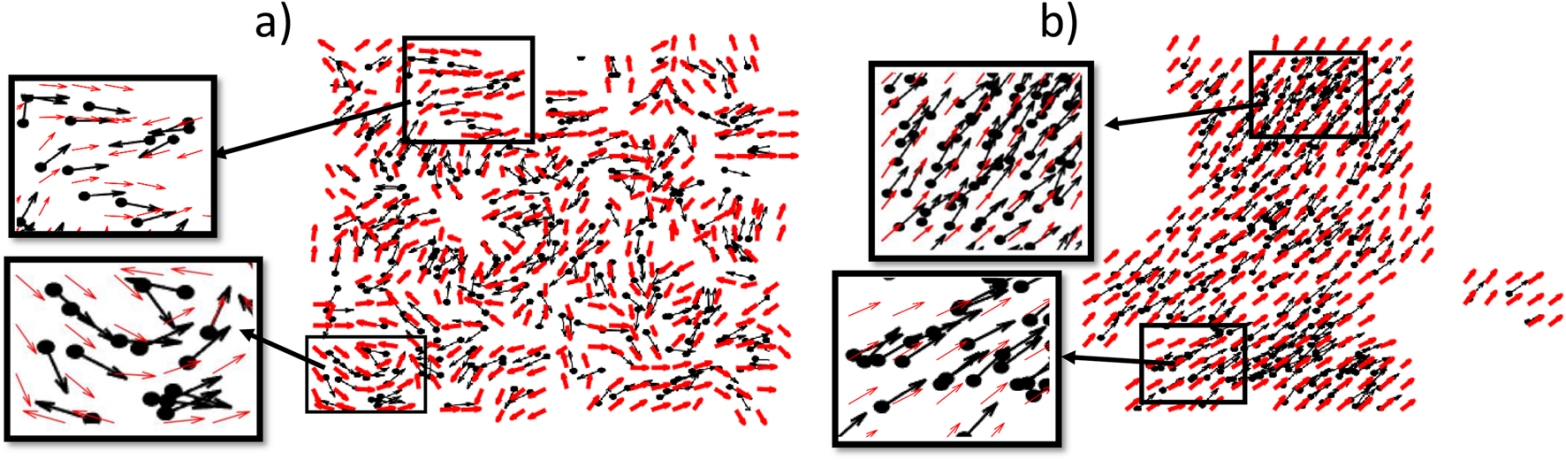
Moving particles (filled ovals) along with PIV vectors (red arrows) at time (**a**) $t=2$, and (**b**) $t=150$ for noise $\eta _0=\frac{\pi }{6}$. Black arrows indicate the headings of respective particles. (**a**) Initially, particles move randomly, producing chaotically directed PIV vectors. (**b**) Particles (as well as PIV vectors) are getting aligned in a specific direction. Note that, if there is no any particle in a grid at either time $t$ or the subsequent time $(t+1)$, the PIV vector is not computed at time $t$, and hence, no arrow is drawn.

**Figure 4:**
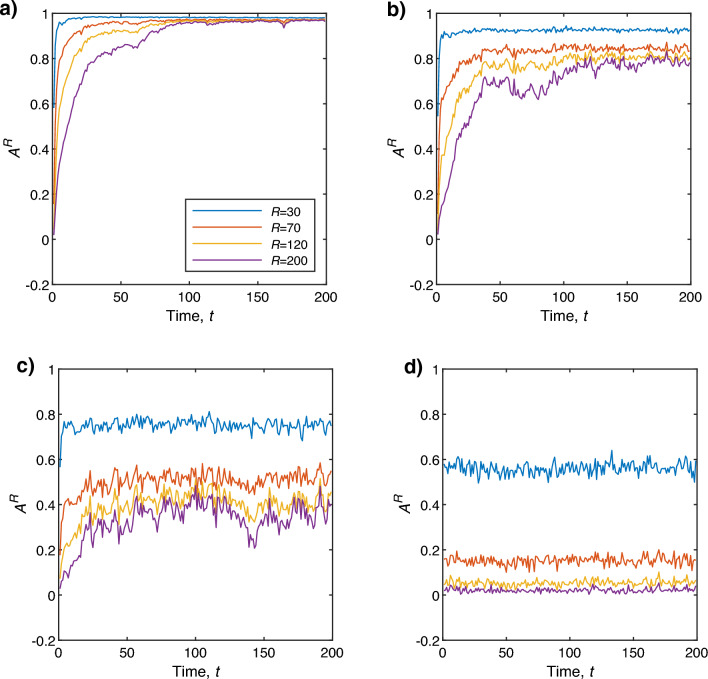
Alignment score $A^R$ for different radius *R* values as a function of time *t* at noise (**a**) $\eta _0=\frac{\pi }{6}$, (**b**) $\eta _0=\frac{3\pi }{6}$, (**c**) $\eta _0=\pi$, and (**d**) $\eta _0=\frac{11\pi }{6}$. The value of *N* for this calculation is set to 300 and the image size is $615\times 615$ pixels. Here the blue, red, yellow, and purple colored lines correspond to $R=30$, $R=70$, $R=120$, and $R=200$ pixels, respectively.

### Averaged normalized velocity

The VM is based on a simple rule where every agent in the swarm aligns its velocity with the average velocity of its neighbors within a certain radius^32,33^ (see “Methods” section “Vicsek model” for a mathematical description of the VM). The alignment of an agent is not perfect, however, there is some noise present in the system. When the intensity of the noise present in the system is low, the system quickly gets aligned in the same direction and moves coherently. But the coherent motion disappears as the noise increases. Particles in the system move randomly at very high noise. This means a phase transition, from ordered motion to disordered states, occurs. This transition can be studied by an order parameter equal to the average normalized velocity having the following form^32^:

2 $$\begin{aligned} v_a(t)=\frac{1}{N}\left| \sum _{i=1}^{N} \frac{\vec {v_i}(t)}{|\vec {v_i}(t)|}\right| , \end{aligned}$$

where *N* is the number of particles present in the system, and $\vec {v_i}(t)$ represents the velocity of the particle *i* at time $t$. If the particles in the system move randomly, the order parameter $v_a$ tends to be zero, and it tends to be 1 if all the particles move in the same direction^32^. This order parameter $v_a(t)$ is defined in such a way that it characterizes the global behavior of the system at time $t$ by incorporating the action of all particles present in the system. In general, coherence may have a length scale, that is, all the particles in the system may not move coherently, but locally some coherence can be observed. To characterize this local behavior, we introduce a variable *R* that portrays the radius of the circle within which the local coherent movement of particles $v_a^{\vec {x_j},R}$ is evaluated:

3 $$\begin{aligned} v_a^{\vec {x_j},R}(t)=\frac{1}{N_j^R(t)}\left| \sum _{i \mathrm { ~s.t.~} |\vec {x_j}-\vec {x_i}|\le R}^{N_j^R(t)} \frac{\vec {v_i}(t)}{|\vec {v_i}(t)|}\right| , \end{aligned}$$

where $\vec {x_j}$ is the center of the circle which is chosen at the initial point $\vec {x_j}$ of a PIV vector *j*, and $N_j^R(t)$ is the number of particles lying within a distance *R* from the PIV vector *j* at time $t$. Point to be noted that the PIV vectors are equally spaced all over the field. We finally take the mean of all local $v_a^{\vec {x_j},R}$’s as follows:

4 $$\begin{aligned} v_a^R(t)=\frac{1}{M} \sum _{j}\left( v_a^{\vec {x_j},R}(t)\right) , \end{aligned}$$

where *M* represents the total number of PIV vectors defined for each image data.

**Figure 5:**
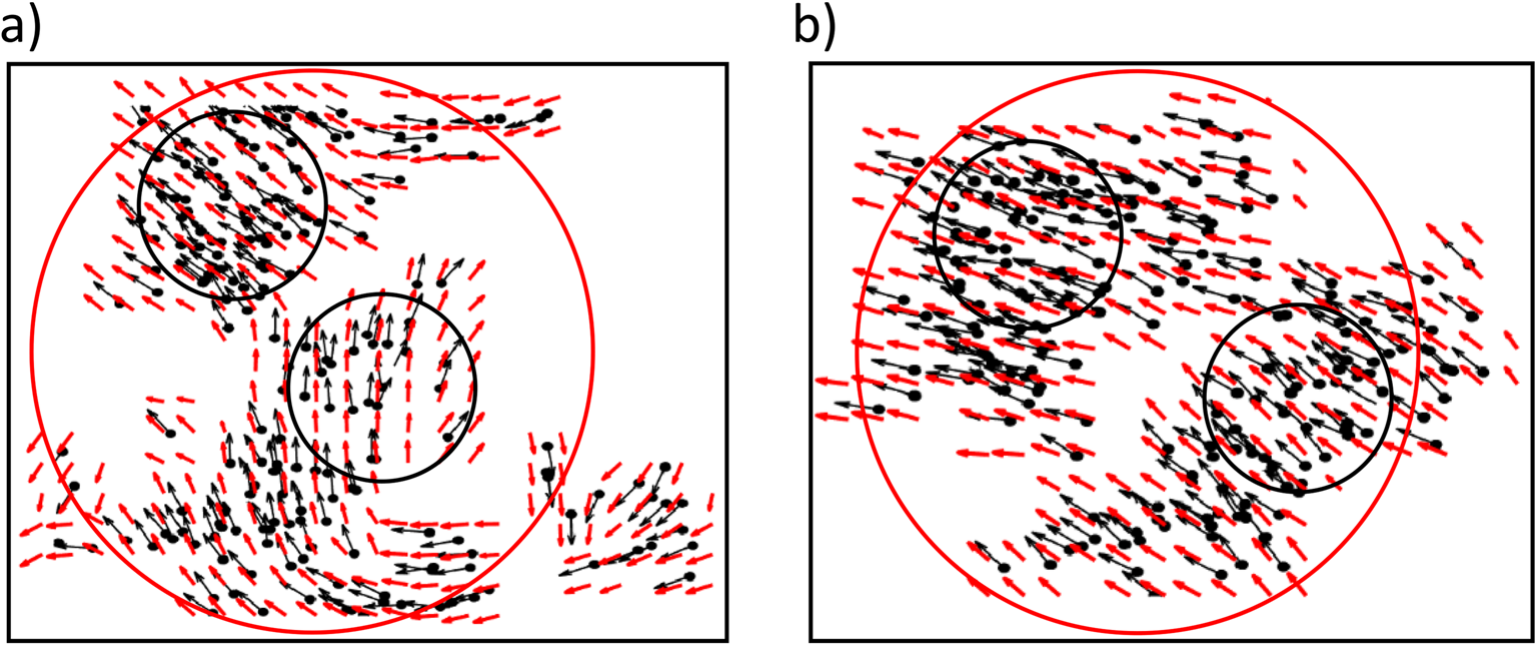
Schematic diagram of *R* dependency of $A^R$. Two clusters of particles (inside black circles) are shown, and within each cluster, all the particles are heading in the same direction. (**a**) The directions of particles within clusters are different. (**b**) The directions of particles within both clusters are the same. Red-colored arrows represent the PIV vectors associated with each cluster. If $A^R$ is computed for the black circles, then in both cases $A^R$ would be very high as within each black circle the direction of the PIV vector and particles are the same. But if $A^R$ is computed for the red circle then for the 1st case $A^R$ will be much lower as the particles are directed in different directions even though for the 2nd case $A^R$ would be close to 1. Note that, if there is no any particle in a grid at either time $t$ or the subsequent time $(t+1)$, the PIV vector is not computed at time $t$, resulting in no arrow.

### Alignment score

As stated in “Particle image velocimetry (PIV)”, PIV uses a statistical measure in the form of cross-correlation to find the best match between two consecutive images, which requires a sufficient amount of data to determine the displacement accurately. Hence, the grid size is chosen so that each grid contains multiple particles^23^, and PIV estimates the mean displacement of a group of particles located within the grid. How well can PIV characterize the underlying particles’ motion? To investigate this issue we introduce a measure named ‘alignment score’ which is the average cosine of the angle between a PIV vector and its neighboring moving particles. The alignment score $A^R(t)$ has the following form:

5 $$\begin{aligned} A^R(t)=\frac{1}{M}\sum _{j=1}^M \frac{1}{N_j^R(t)}\sum _{i \mathrm { ~s.t.~} |\vec {x_j}-\vec {x_i}|\le R} ^{N_j^R(t)}\frac{\vec {v_j^t}\cdot \vec {w_i^t}}{|\vec {v_j^t}| |\vec {w_i^t}|}, \end{aligned}$$

where $\vec {v_j^t}$ and $\vec {w_i^t}$ denote the *j*th PIV vector and the velocity vector of particle *i*, respectively, at time *t*, and ‘$\cdot$’ represents the dot product between the two vectors. The second sum in Eq. (5) is taken over all particles *i* located within a distance *R* from the initial point of the PIV vector *j* at time *t*. The value of $A^R$ lies between − 1 and 1, where 1 means that all the particles within the circle are perfectly aligned to the PIV vectors, 0 means that the particles have no tendency to be aligned with the PIV vectors, and − 1 means that the particles move in the exact opposite direction of PIV vectors (see Fig. 2 for a visual illustration). Whenever the value of $A^R$ is greater than zero, it means that PIV vectors have some alignment on average with the particle motion.

**Figure 6:**
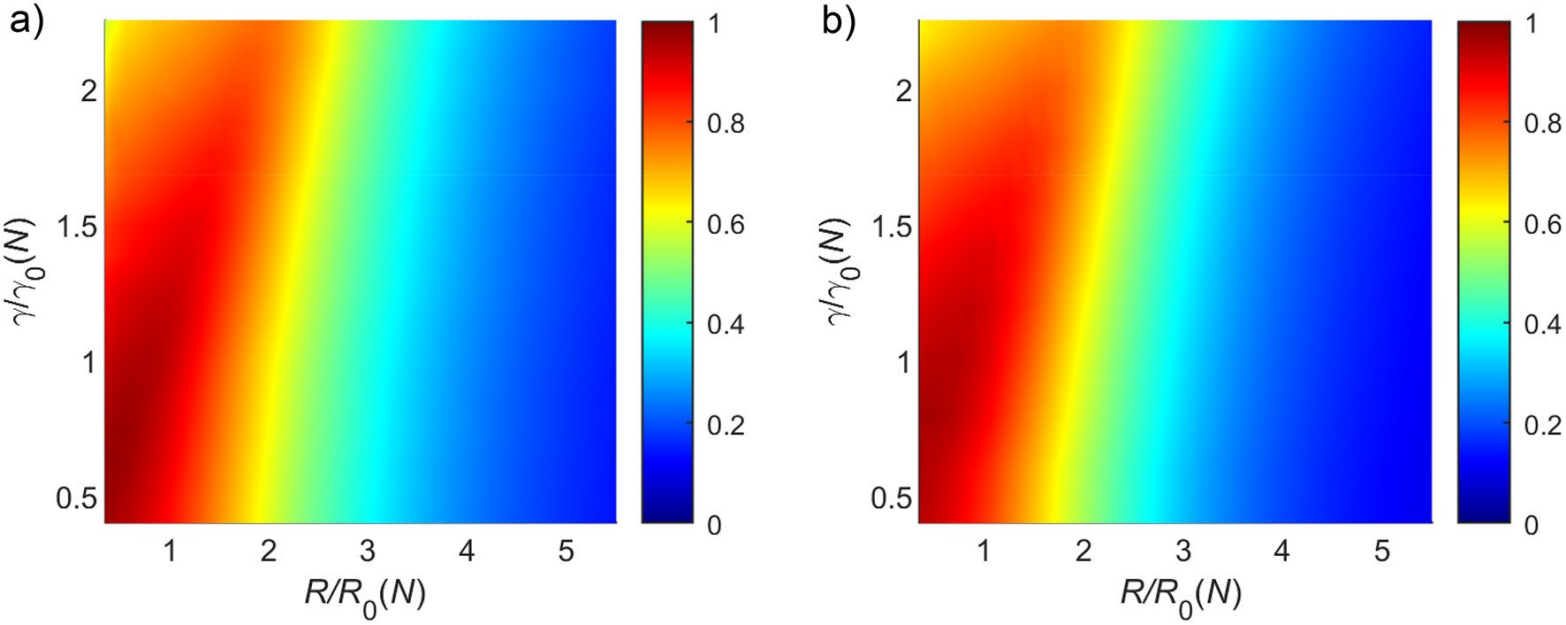
$A^R$ landscape at $\eta _0=\frac{11\pi }{6}$ (high noise) as a function of normalized PIV grid size, $\frac{\gamma }{\gamma _0(N)}$ and normalized radius, $\frac{R}{R_0(N)}$ for (**a**) $N = 100$ (corresponding to $\gamma _0 = 61.5$ pixels and $R_0 = 34.7$ pixels), (**b**) $N=300$ ($\gamma _0=35.5$, $R_0=20$). It is apparent that $A^R$ values of close to 1 are obtained when both $\frac{\gamma }{\gamma _0(N)}$ and $\frac{R}{R_0(N)}$ are close to 1. When both $\frac{\gamma }{\gamma _0(N)}$ and $\frac{R}{R_0(N)}$ exceed 1, the value of $A^R$ begins to decrease. A similar trend in $A^R$ values has been found for different *N* values (see also Supplementary Figure 4 for $N=200,500,700, \text{ and } 1000$. The value of $A^R$ decreases along the direction normal to the slope $\frac{d\gamma }{dR}\sim 2$ approximately in this normalized plane, which implies that the radius *R* becoming two times larger is equivalent to an increase in $\gamma$ four times. This is due to the geometrical reason that one circle of radius *R* is involved in the grid of $\gamma \times \gamma$. The intervals along the horizontal and vertical axes are not the same for every *N* since the values of $\gamma _0$ and $R_0$ vary with *N* with a series of different, discrete $\gamma$ values. The figures were plotted using the MATLAB ‘shading interp’ function to interpolate the data points or grids for different number of grids.

## Results

Since, in simulating the VM, we know the positions and velocities of individual particles at each time instance, we can use these trajectories to test the performance of PIV in capturing the single particle’s motion. We mimic images of the particles by plotting markers representing agents at each particle location which are oriented in the direction of particle motion. The PIV technique is applied to the images, and we can compare the velocities computed by PIV to the velocities known from the trajectories using the alignment score (defined in Eq. 5).

Figure 3 represents some snapshots of moving particles (black arrows with filled ovals) along with PIV vectors (red arrows) for noise $\eta _0=\frac{\pi }{6}$ at time (a) $t=2$, and (b) $t=150$. In the VM, the initial direction and position of particles are chosen randomly. Consequently, $t=2$ is too early to observe a global feature of moving particles. Since the particles within two nearby PIV grids are not aligned with one another, the PIV vectors associated with nearby grids are also not aligned with one another. Hence, at time $t=2$, PIV vectors do not exhibit global coherence (Fig. 3a). When looking closer at the PIV vectors and their nearby particles (insets of Fig. 3a), some coherence in the orientations of particles is observed at such smaller length-scales, and the PIV vectors appear to be aligned with their nearby particles. As time passes, the coherent movement of particles begins to be visible (Fig. 3b). At this time, small groups of particles are observed which are directed in the same direction. Hence, the direction of PIV vectors placed within a cluster of particles will be the same as the direction in which the particles are heading. As more time passes, all the particles in the system start to move in the same direction, and the whole system acts as a single cluster that produces the PIV vectors directed in the same direction at this noise level. Note that in Fig. 3b, even though there are the same amount of particles in each image, there can be some vacant regions. The PIV vector is not computed and not drawn at a given time $t$ if there is no any particle in a grid at either the time $t$ or the consecutive time $(t + 1)$.

How precisely can the PIV vectors dictate the movement of neighboring particles? Figure 4 shows the alignment score $A^R$, defined in “Alignment score”, as a function of time $t$ at noise level (a) $\eta _0=\frac{\pi }{6}$, (b) $\eta _0=\frac{3\pi }{6}$, (c) $\eta _0=\pi$, and (d) $\eta _0=\frac{11\pi }{6}$. It appears that at low noise $(\eta _0=\frac{\pi }{6})$, $A^R$ quickly converges to its maximum value 1 for small *R* values, and the $A^R$ value decreases as the radius *R* rises at early times (Fig. 4a). The global coherence of particles takes some time to form since, according to the VM’s interaction rule, particles cannot interact globally straight away as shown in the $v_a$ vs time $t$ figure (see Supplementary Figure 1). This delay in the coordinated movement of agents is a very common phenomenon in nature as information cascade among group members requires some time. However, the local coherent motion of the particles can be seen as illustrated in Fig. 5a. If the values of *R* are set to less than the size of the local collectives (black circles in Fig. 5a), then $A^R$ will be very high ($\sim 1$). Conversely, if the values of *R* are set to higher than the size of the local collectives (red circle in Fig. 5a), the value of $A^R$ decreases as the particles oriented in different directions are considered. Since PIV measures the average displacement of particles located within a grid, hence average cosine of the angle between a PIV vector and its neighboring particles decreases as more randomly moving particles are considered. On the contrary, if all the particles in the system move in the same direction ($v_a\sim 1$ (Supplementary Figure 1)), then the value of $A^R$ will always be $\sim 1$ regardless of the values of *R* (Fig. 5b) as the direction of all PIV vectors will also be the same as the particles. Therefore, once the system reaches global coherence, $A^R$ does not depend on *R* to such a considerable level. As the noise $\eta _0$ increases the value of $A^R$ decreases (Fig. 4b–d). The transition from order to disorder is found to be at a noise level of around $\pi$ (see Supplementary Figure 2). In the original paper by Vicsek^32^, it was reported that $v_a\approx 0.5$ at noise $\eta _0 =3$ for $N=100$, and $\eta _0$ is slightly less than 3 for $N=400$. Our model has slightly different parameter values such as the box size *L*, interaction domain *R*, *N*, and we consider our finding to be consistent with Ref.^32^. At this noise level, it was found that the alignment score $A^R$ fluctuates over time (see Supplementary Figure 3c), which indicates that transient coherent and incoherent motions are intermingled. At very high noise $(\eta _0=\frac{11\pi }{6})$ $A^R$ is close to zero as the noise dominates the system and particles move randomly (Fig. 4d). It has been found that even for very high noise $(\eta _0=\frac{11\pi }{6})$, $A^R$ is relatively high $(\sim 0.5 )$ for $R=30$. Thus at high levels of noise, it is important to choose *R* such that the number of particles within the radius *R* is not too large as well as the size of grids to evaluate PIV vectors in order to capture and the underlying particle’s motility and properly characterize the accuracy of PIV using the quantity $A^R$.

**Figure 7:**
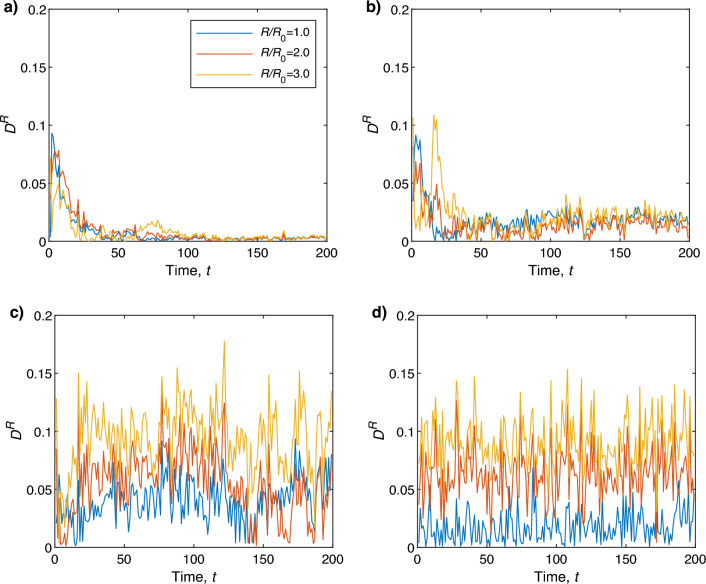
$D^R$ as a function of time $t$ for different *R* values at noise (**a**) $\eta _0=\frac{\pi }{6}$, (**b**) $\eta _0=\frac{3\pi }{6}$, (**c**) $\eta _0=\pi$, and (**d**) $\eta _0=\frac{11\pi }{6}$. For this calculation, the grid size $\gamma$ and the value of *N* are set to 64 pixels and 300, respectively. Here the blue, red, yellow, and purple colored lines correspond to $R/R_0=1$, $R/R_0=2$, and $R/R_0=3$, respectively.

**Figure 8:**
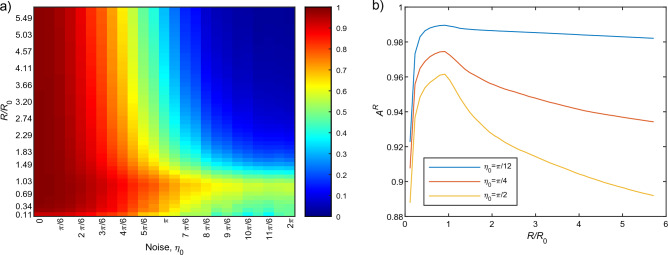
(**a**) Alignment score $A^R$ landscape as a function of normalized radius $R/R_0$ and noise $\eta _0$ for $N=300$ and $\gamma =64$ $(=0.986\gamma _0)$ pixels. The $A^R$ can take a value between − 1 and 1 in principle, but the smallest observed value was 0.0205 so that we plot $A^R$ landscape for $A^R ~\in [0:1]$. (**b**) $A^R$ as a function of normalized radius $R/R_0$ for low and moderate noise levels.

How to determine the optimal *R* and the grid size in the PIV estimation at high noise cases? PIV measures the average displacement of particles located within a grid of size $\gamma \times \gamma ~(\gamma \in {\mathbb {R}})$. Hence, PIV cannot precisely measure the velocities when there are several particles in a PIV grid because, in the case of very high noise, particles move randomly. In principle, if a PIV grid contains only a single particle, then PIV should be able to characterize the motion of that particle even for very high noise cases. Let $\gamma _0(N)$ represents the typical size of the PIV grid for a given number of particles *N*, which contains only a single particle on average, which has the following form:

$$\begin{aligned} \gamma _0(N)=\sqrt{\frac{\Gamma \times \Gamma }{N}}, \end{aligned}$$

where $\Gamma$ represents the length of the image data. Likewise, under the assumption of uniform distribution, $R_0(N)=\gamma _0(N)/\sqrt{\pi }$ is the radius size of the circle centered at each pixel for a given number of particles *N*, having only a single particle on average. Figure 6 represents the $A^R$ landscape as a function of normalized PIV grid size, $\frac{\gamma }{\gamma _0(N)}$, and normalized radius, $\frac{R}{R_0(N)}$ for (a) $N=100$ and (b) $N=300$ with $\eta _0=\frac{11\pi }{6}$ (high noise case). The $A^R$ values get closer to unity when $\frac{\gamma }{\gamma _0(N)}$ and $\frac{R}{R_0(N)}$ are proximate to unity, which results from the fact that, on average, the PIV grid of size $\gamma$ contains a single particle, and $A^R$ estimation is also carried out with a small circle within which only a single particle exists on average. Consequently, the PIV vector captures the motion of the particle lying inside that grid appropriately. On the other hand, a $\frac{\gamma }{\gamma _0(N)}$ value greater than 1 means that, on average, a PIV grid of size $\gamma$ may contain multiple, randomly-moving particles, hence the $A^R$ value decreases (for all *R* values) as the value of $\frac{\gamma }{\gamma _0(N)}$ increases over 1, at such high noise levels where the particles have no collectives. Likewise, an $\frac{R}{R_0(N)}$ value of greater than 1 may signify that several randomly-moving particles are being taken into account, which results in low $A^R$ values. A universal trend in $A^R$ values has been found for different *N* values (see Supplementary Figure 4).

To characterize the local coherent movement of particles we defined $v_a^R(t)$ in Eq. (4), where the headings of particles located within a distance of *R* from the initial point of the PIV vector *j* were considered. The same quantity can also be used to check the alignment of PIV vectors located within a circle of radius *R*. Now we have two different $v_a^R(t) ~(\in [0,1])$, one is for the moving particles (self-propelled particles (SPPs)), we denote it by $v_a^{R,\textrm{SPP}}(t)$, and the other one is for the PIV vectors which is denoted by $v_a^{R,\textrm{PIV}}(t)$. Hence the difference defined by

$$\begin{aligned} D^R(t)= \left| v_a^{R,\textrm{SPP}}(t)- v_a^{R,\textrm{PIV}}(t)\right| \end{aligned}$$

quantifies the difference in coherent movement of SPPs and alignment of PIV vectors located within a distance of *R*. $D^R$ can take a value between 0 and 1 and, for example, $D^R\sim 0$ means that both the PIV vectors and nearby particles are directed in the same direction. Figure 7 shows $D^R$ as a function of time *t* for different $R/R_0$ values at noise (a) $\eta _0=\frac{\pi }{6}$, (b) $\eta _0=\frac{3\pi }{6}$, (c) $\eta _0=\pi$, and (d) $\eta _0=\frac{11\pi }{6}$. Here the value of *N* is set to 300, and the grid size $\gamma$ corresponds to $\gamma =0.986\gamma _0$. Regardless of normalized radius $R/R_0$ values, $D^R$ rises as the noise level increases (Fig. 7). Here, $R/R_0 \simeq 1$ denotes that there is typically just one particle present within the circle (on average under uniform distribution), and more noticeable is that, the value of $D^R$ increases for $R/R_0=1$ faster than for other $R/R_0$ values as the noise level $\eta _0$ gets larger. Here, the PIV grid size $\gamma$ was set to 64 pixels, which means the distance between two neighboring PIV vectors is 64 pixels and $R/R_0=1$ corresponds to 35.5 pixels in the current settings. Hence the circle of radius $R\le R_0$ pixels centered at the initial point of a PIV vector contains only one PIV vector that produces $v_a^{R,\textrm{PIV}}=1$ by definition. However, that circle may contain more than one SPP. Here $v_a^{R,\textrm{SPP}} \approx 1$ for low noise and relatively longer time as the particles within the circle quickly get aligned, resulting in a small $D^R$ over the long duration (Fig. 7a). As the noise increases, $v_a^{R,\textrm{PIV}}$ remains the same (i.e., 1) for $R=R_0$, but the particles inside the circle begin to disintegrate which lowers the values of $v_a^{R,\textrm{SPP}}$. As a result, $D^R$ increases as the noise increases for $R=R_0$ (Fig. 7b–d). But if the value of *R* is set larger than $R_0$ (larger than the PIV grid size $\gamma$), the circle contains multiple PIV vectors. Consequently, the value of $v_a^{R,\textrm{PIV}}$ decreases as the noise increases. Hence, $D^R$ also decreases for $R/R_0>1$, because of the difference between the small numbers. Similar results have been found for different grid sizes and normalized radii.

The landscape of $A^R$ as a function of noise $\eta _0$ and normalized radius $R/R_0$ is shown in Fig. 8a. The alignment score $A^R$ was computed as the average taken over 30 trials (initial conditions) and the time *t* was set to 300, which means PIV (for $A^R$ computation) was applied on images at time $t=299$ and $t=300$ only. For all noise levels, it can be seen that $v_a$ stabilizes within this time frame (see Supplementary Figure 5). It was found that at the low level of noise $(\eta _0\le \pi /2)$ $A^R$ is very high ($\approx 1$) (see Fig. 8a). At low noise levels, particles tune very quickly in the same direction. Consequently, the value of $A^R$ does not rely greatly on radius *R*. As the noise increases, particles move more randomly instead of coherently. Consequently, the value of $A^R$ drops as the noise increases. On the other hand, a smaller radius means only the close particles, close to the PIV vector, are taken into consideration. Hence, even at relatively higher noise, a relatively high $A^R$ was found for $R/R_0\lesssim 1.0$. As the radius increases, distant particles begin to be incorporated that resulting in low $A^R$ values $(\approx 0)$. It may be noted worthy that a peak in $A^R$ values near $R/R_0 \approx 1$ can be observed, which is pronounced for the noise interval $\pi \le \eta _0\le 2\pi$. The reason of this peak near $R/R_0 \approx 1$ lies in the PIV computation. The PIV grid size $\gamma$ was set to 64 pixels, which means a PIV vector quantifies the average motion of the particles located within a box of size $64 \times 64$. The maximum radius of the circle that fits into the box is $\approx 36$ pixels. Some particles that the PIV vector depends on are ignored if the value of *R* is set lower than 36 pixels. In contrast, if we increase *R* over 36 pixels, we start to take into account those particles from which the PIV vector is independent. $A^R$ would therefore reach its maximum at $R\approx 36$ pixels. It should be noted that the value of $R_0$, the typical radius size of a circle that contains only a single particle on average, is 35.5 pixels. Hence, $A^R$ reaches to its maximum at $R/R_0\approx 1$. Figure 8b demonstrates that even in low and moderate noise cases, this characteristic of $A^R$ remains unchanged as one can find a peak near $R/R_0\approx 1$.

In principle, our comparison of PIV performance at different noise levels showed that PIV has appropriate length scales to detect the underlying agents’ motility dependent on the noise levels and works best whenever single particles are isolated from one another on average in the alignment grid. This should also be relevant to what timescale the series of the images are acquired. For example, an overdamped Langevin equation is represented by its difference form as $x(t+\Delta t)=x(t)+\frac{1}{\gamma } \{ F(x)+ \xi (t)\}\Delta t$ where *x*, *F*(*x*), $\gamma$, and $\xi (t)$ denote the coordinate of the system, the mean force, friction coefficient, random force, respectively. That is, the difference form of the overdamped Langevin equation tells us that random force exerted on the coordinate *x*(*t*) is regarded as being proportional to time increment $\Delta t$. The time increment of Vicsek model $\Delta t$ and the recording time step were both unity in our simulation, and high noise in Vicsek model (e.g, $\eta _0= 11\pi /6$) corresponded to “high temperature” at which the movements of particles are subject to significant changes in their directions within the time unit $\Delta t$. What we showed is that, especially in such case, the desired grid size corresponds to the situation that only one agent exists on average within each grid. In turn, low or moderate noises in Vicsek model corresponds to “low temperatures”. In such cases, within the time unit $\Delta t$ the movements of particles are less subject to large changes in their directions. Low or moderate noises in Vicsek model might mimic “a shorter timescale” than the timescale when a system finds the substantial changes in their configuration. Supplementary Figure 6 shows the effect of sampling rate of the images on the time averaged alignment score ${<}A^R{>}$ at intermediate noise $\eta _0= \pi /2$ in which the recording time step $\tau$ is varied while the time increment of the Vicsek model $\Delta t$ is unity. The figure demonstrates that, as the recording time step $\tau$ increases, the PIV analysis misses the details about the flow field, leading to low ${<}A^R{>}$ values. It may be noted worthy that the decreasing rate of the average $A^R$ with respect to $\tau$ tends to get more pronounced when the radius *R* is deviated from $R_0$.

## Conclusion

In this study, we have scrutinized the use of PIV in assessing the motion of agents at a variety of noise levels. As is known for the VM, the system undergoes a transition from ordered to disordered phase as the increase of the noise value. As expected, the PIV vector aligns well with the motion of agents when the noise level is low, resulting in global alignment. When the noise level becomes higher, different behavior in PIV vectors is observed. PIV can generally pick up the local agent motion, and alignment scores are high at low radius even when the noise level is high. At higher radii, however, the alignment score drops because a PIV vector cannot characterize the motion of agents that are far away at high noise levels. We have also computed the alignment score using the optical flow (OF) constraint scheme^23^, which measures the motion of individual pixels, at different noise levels. It has been observed that the alignment scores using OF data are almost identical to that using PIV data (see Supplementary Figure 7). Theoretically, PIV can be considered as the coarse-grained scheme of OF. Hence one may use OF to get deep-rooted information of the flow field. Nevertheless, one should also keep in mind that computationally OF, which computes displacement vector pixel-wise, is not as efficient as PIV. Hence depending on individual research perspectives, one may choose a suitable scheme.

An interesting future area of study would be to characterize cell motion and its relation to PIV at a more coarse-grained level in these parameter ranges. This is one of the forthcoming subjects to be addressed along this line. Another important subject is concerning how to design and choose the appropriate grid size to compute the underlying agents dynamics in real applications under the constraint one cannot access the underlying ground truth motility of the agents. As Fig. 6 shows, as the grid size $\gamma$ decreases and approaches to $\gamma _0$, it was shown that the PIV vectors can capture the underlying agents dynamics irrespective of the noise level. In practice, while one may not be able to access the value of $\gamma _0$, we expect that one can choose the appropriate grid size by checking the convergence of resultant PIV vectors in real applications. In addition, the forthcoming subjects to be solved for real applications such as fluorescence cell images are, for example, cell division, mortality, and also optical artifacts like out-of-focus effects, noise during image acquisition, which affect the estimation of optimal grid size to retrieve cell motility. These are some of the imminent issues to be addressed in the future.

## Methods

### Vicsek model

A flocking model was proposed by Vicsek et al.^32^ to study the phase transition of a group of collectively moving particles. The model consists of a two-dimensional square box of length *L*, and *N* self-propelled particles move within the box with periodic boundary conditions^34^. For simplicity, it was assumed that all the particles move with the same constant speed $v_0$ and can move in any direction. The initial (at time $t=0$) positions of particles are distributed randomly in the square box, and the orientations are also chosen randomly within $[0,2\pi ]$. The position of the $i-$th particle at time $t+1$ is denoted by $\vec {r_i}^{t+1}=(x_i(t+1),y_i(t+1))$ and is given by the following discrete-time stochastic system:

$$\begin{aligned} \vec {r_i}^{t+1}=\vec {r_i}^{t}+\vec {v_i}^{t}\Delta t, \end{aligned}$$

where $\vec {v_i}^{t}=(v_0 \cos \theta _i (t),v_0 \sin \theta _i(t) )$ is the velocity of the particle $i$ at time $t$, and $\theta _i(t)$ be its orientation. At each time step the orientation angle of particle $i$ is computed as the average of the orientations of the particles which are located within a circle of radius $r$ centered at $\vec {r_i}^{t}$ including the particle $i$ itself with some random perturbation added. Hence the direction update rule of the particle $i$ is as follows:

$$\begin{aligned} \theta _i(t+1)=<\theta _i(t)>_r+\Delta \theta . \end{aligned}$$

Here $\Delta \theta$ is the random perturbation of the system distributed uniformly in the range $[-\frac{\eta _0}{2},\frac{\eta _0}{2}]$, where $\eta _0$ is considered as a temperature-like parameter. Suppose ${\mathcal {N}}_i(t)$ be the set of all neighboring particles (within radius $r$) of $i$ at time $t$ including itself. Then the average direction is given by^35^:

$$\begin{aligned} <\theta _i(t)>_r=\text {arctan}\left[ \frac{\sum _{j\in {\mathcal {N}}_i(t)}\sin \theta _j(t)}{\sum _{j\in {\mathcal {N}}_i(t)}\cos \theta _j(t)}\right] \end{aligned}$$

In this paper, the value of the box size *L* is set to be 5 arb. units (615 pixels), and the constant speed of particles $v_0$ being 0.03 arb. units (3.7 pixels per frame). The value of the interaction radius $r$ is set to be 0.5 arb. units (61.5 pixels). Results shown in “Results” are for $N = 300$ besides Fig. 6. But it was found that in the range of $100 \le N \le 2000$, the alignment score $A^R$ was found to be in similar order (see Supplementary Figure 8).

### PIV parameters

PIV technique relies on the intensity variation of image data. In this study, gray-scale images are generated by plotting black-filled ovals at each particle location. Hence, a pixel with an intensity of 0 represents a part of a particle, whereas an intensity value of 255 represents the background. The size of a particle is set to be $8 \times 6$ pixels, where 8 indicates the length of the major axis and 6 the length of the minor axis. Since PIV estimates the mean displacement of particles, hence it is directly related to the size of the interrogation zone (grid size, $\gamma$). It was found that the alignment score $A^R$ remains very consistent as long as the particle size is considerably less than $\gamma$ (see Supplementary Figure 9). It is easily understandable that PIV is unable to detect intensity variation when a particle covers the entire PIV grid. Hence, $A^R$ should decrease if the particle size gets much closer to the grid size, $\gamma$. For the statistical purpose, the grid size is chosen large enough so that it includes a significant number of particles^36,37^. Hence, the grid size was set to be $64 \times 64$ pixels, unless stated otherwise. The step size which defines the interrogation/search zone was set to be $32 \times 32$ pixels ($50\%$ of the grid size). That means the size of the search zone was set to be $96 \times 96$ pixels. It was found that the grid and step size do not have much effect on the alignment score $A^R$ for a low level of noise (not shown). Finally, the images were analyzed using the particle image velocimetry (PIV) technique with PIVlab^38^, and the $A^R$ computation is carried out by excluding the outlier PIV vectors using the ‘vector validation’ function.

## Supplementary Information

Supplementary Information.

## Data Availability

The data that support the findings of this study are available from the corresponding author upon request.

## Code availability

The code employed to create all simulations is accessible at https://zenodo.org/record/8174506.
