# Supplementary material for: Comparison of particle image velocimetry and the underlying agents dynamics in collectively moving self propelled particles
Source: Sci Rep. 2023 Aug 2;13:12566. doi: 10.1038/s41598-023-39635-z (PMC10397335; doi:10.1038/s41598-023-39635-z)
Supplement: Supplementary file 1 — Supplementary Information. [file 41598_2023_39635_MOESM1_ESM.pdf]

SUPPLEMENTARY MATERIALS FOR ‘COMPARISON OF PARTICLE IMAGE VELOCIMETRY AND THE UNDERLYING AGENTS DYNAMICS IN COLLECTIVELY MOVING SELF PROPELLED PARTICLES’

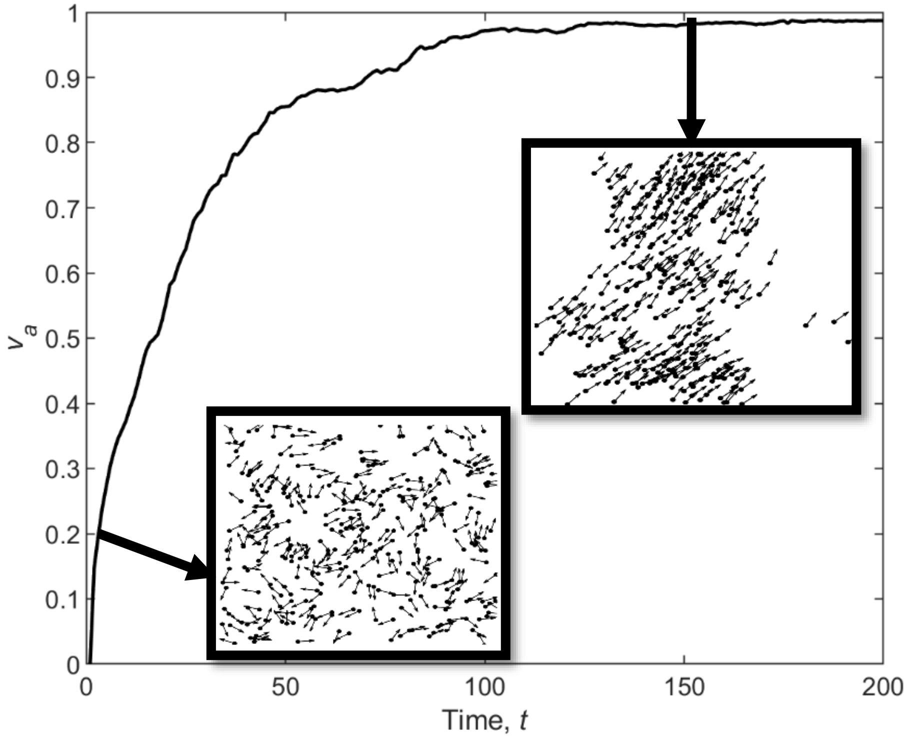

Supplementary Figure 1. Average normalized velocity v_a at noise $\eta_0 = \frac{\pi}{6}$. It appears that v_a increases with time and it approaches its maximum near $t = 150$.

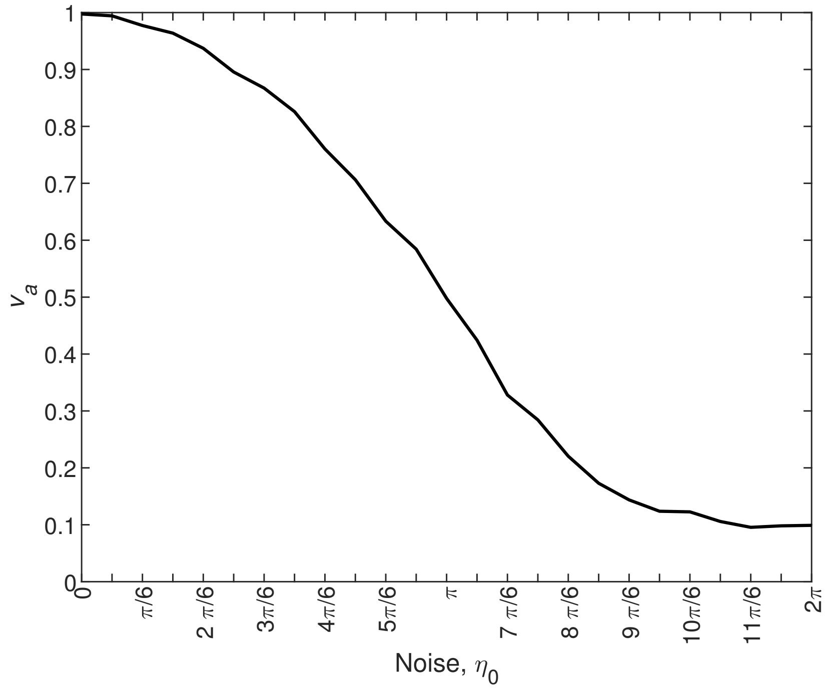

Supplementary Figure 2. v_a as a function of noise η_0 for $N = 300$. It was found that the system changes from the ordered state to the disordered state at a noise level of around π. v_a was computed as the average taken over 50 trials (different initial conditions).

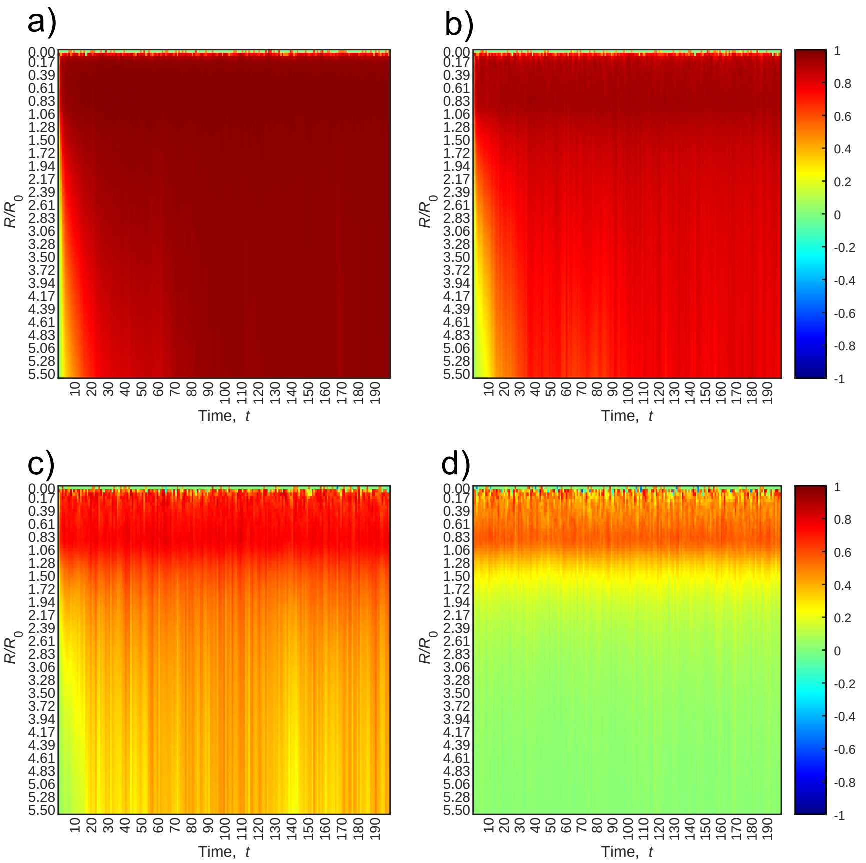

Supplementary Figure 3. Alignment score A^R landscape as a function of time t and normalized radius R/R_0 at noise (a) $\eta_0 = \frac{\pi}{6}$, (b) $\eta_0 = \frac{3\pi}{6}$, (c) $\eta_0 = \pi$, and (d) $\eta_0 = \frac{11\pi}{6}$ using PIV data. The alignment score A^R has been found to decrease when the noise η_0 increases for a fixed value of R/R_0. One can clearly see that the alignment score A^R decreases when the relative radius R/R_0 increases above 1 at the beginning of time, especially for the relatively low noise levels (a and b). For (c), it is not so apparent as the cases of (a) and (b), and eventually at the highest noise (d), only the short-range $R/R_0 \lesssim 1$ where the number of agents is less than unity, the value of A^R is close to unity.

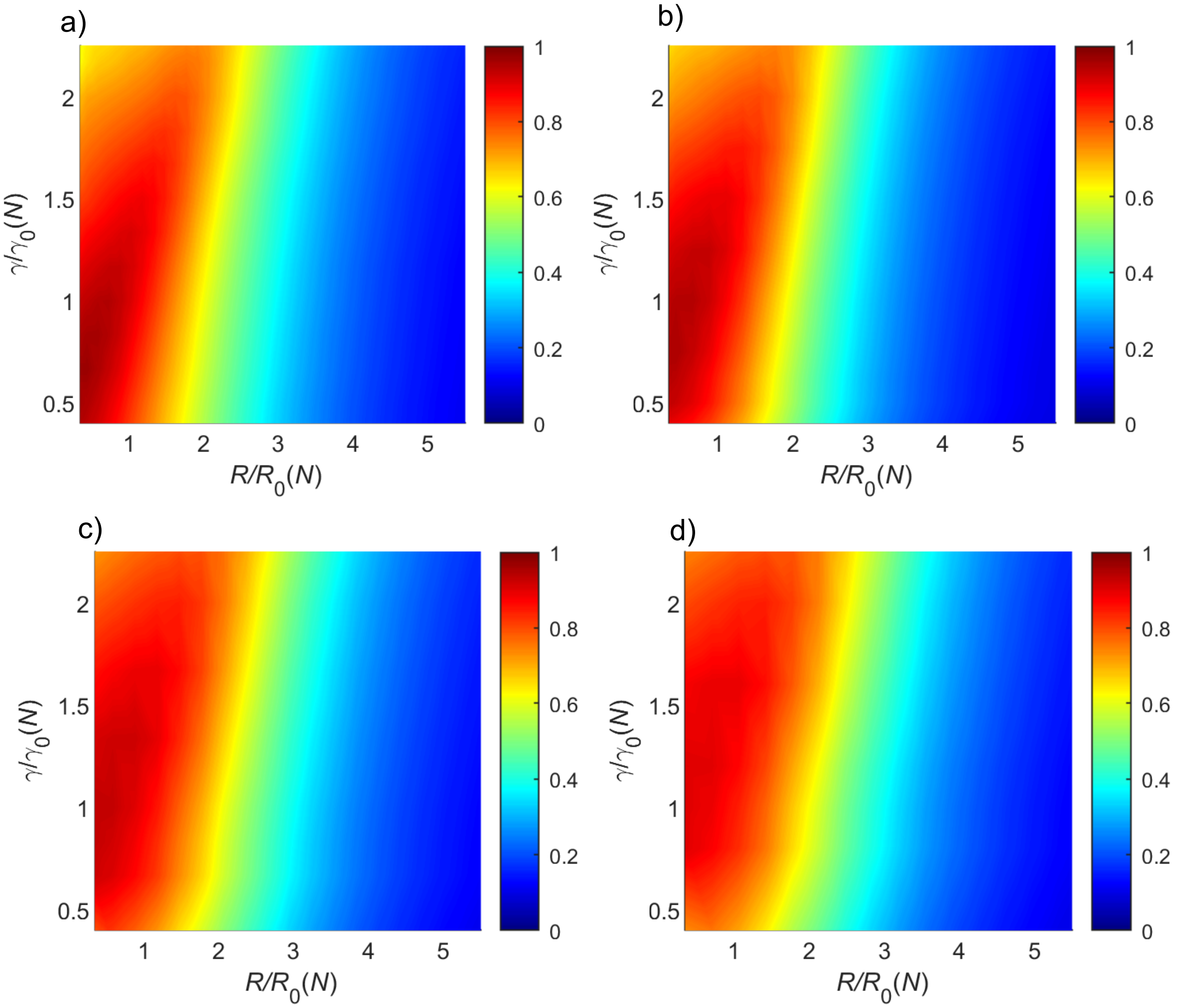

Supplementary Figure 4. A^R landscape at $\eta_0 = \frac{11\pi}{6}$ (high noise) as a function of normalized PIV grid size, $\frac{\gamma}{\gamma_0(N)}$ and normalized radius, $\frac{R}{R_0(N)}$ for (a) $N = 200$ (corresponding to $\gamma_0 = 43.5$ pixels and $R_0 = 24.5$ pixels), (b) $N = 500$ ($\gamma_0 = 27.5, R_0 = 15.5$), (c) $N = 700$ ($\gamma_0 = 23, R_0 = 13$), (d) $N = 1000$ ($\gamma_0 = 19.5, R_0 = 11$). See also the caption of Figure 6 (main text).

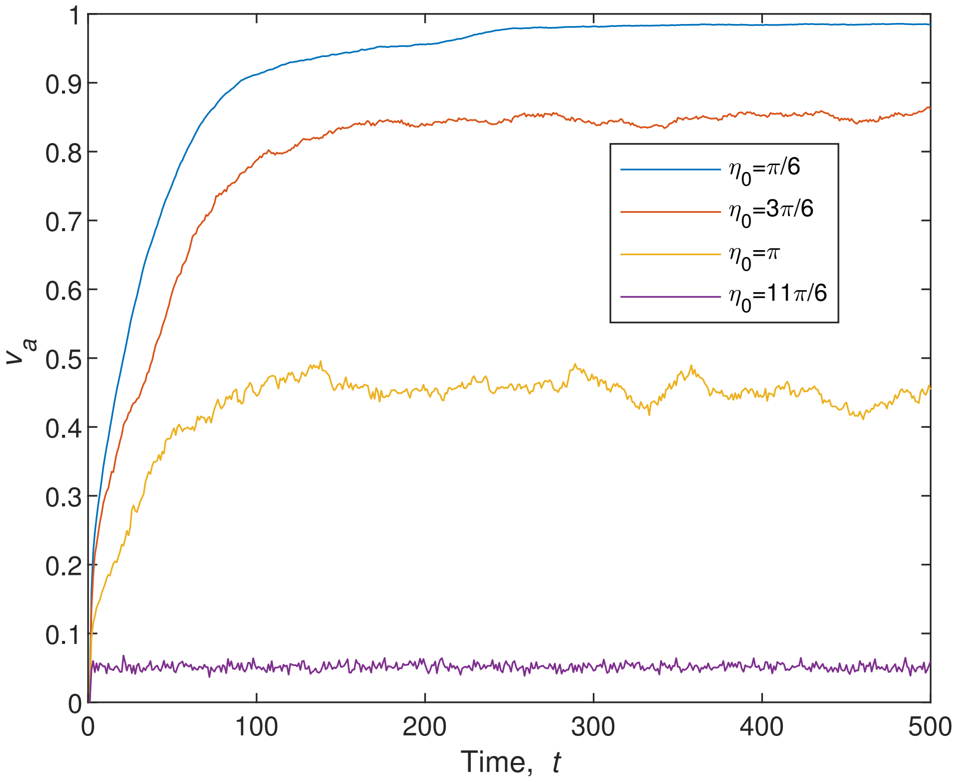

Supplementary Figure 5. Average normalized velocity v_a as a function of time t for different noise values. Here v_a is computed as the average taken over 30 different initial conditions. v_a generally tends to decline as the noise level rises. Additionally, it becomes stable at around $t = 300$.

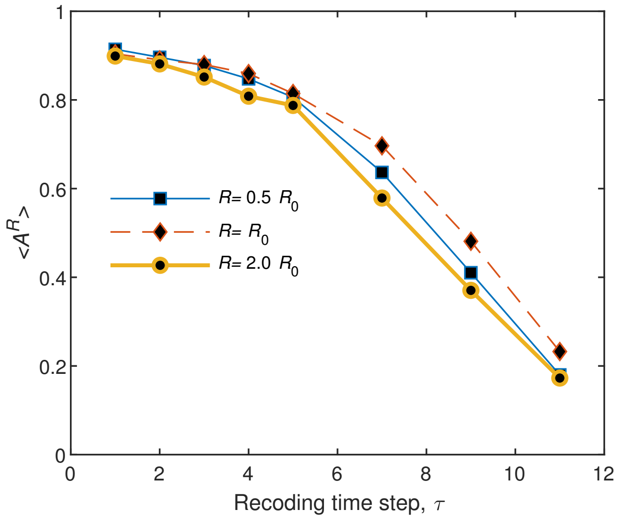

Supplementary Figure 6. Average alignment score, $<A^R>$ for different τ and R values at noise $\eta_0 = \pi/2$. The recording time step τ means that only every τ time steps' information is used to construct the images for the PIV analysis.

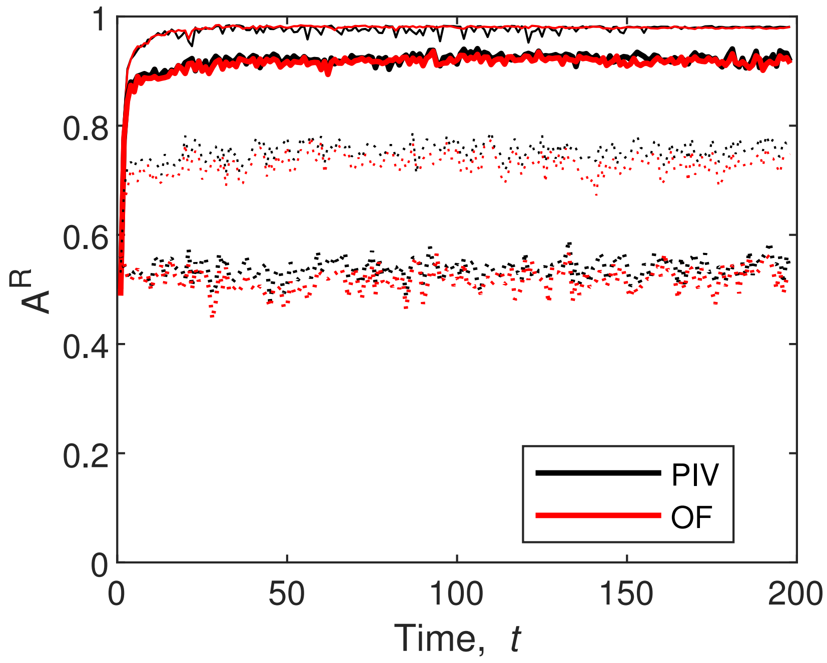

Supplementary Figure 7. The time evolution of the alignment score A^R for different noise levels $\eta_0 = \frac{\pi}{6}$ (thin solid lines), $\eta_0 = \frac{\pi}{2}$ (thick solid lines), $\eta_0 = \pi$ (thin dotted lines), $\eta_0 = \frac{11\pi}{6}$ (thick dotted lines) using PIV and OF data. It appears that the alignment score A^R for both PIV and OF data is almost the same regardless of noise levels.

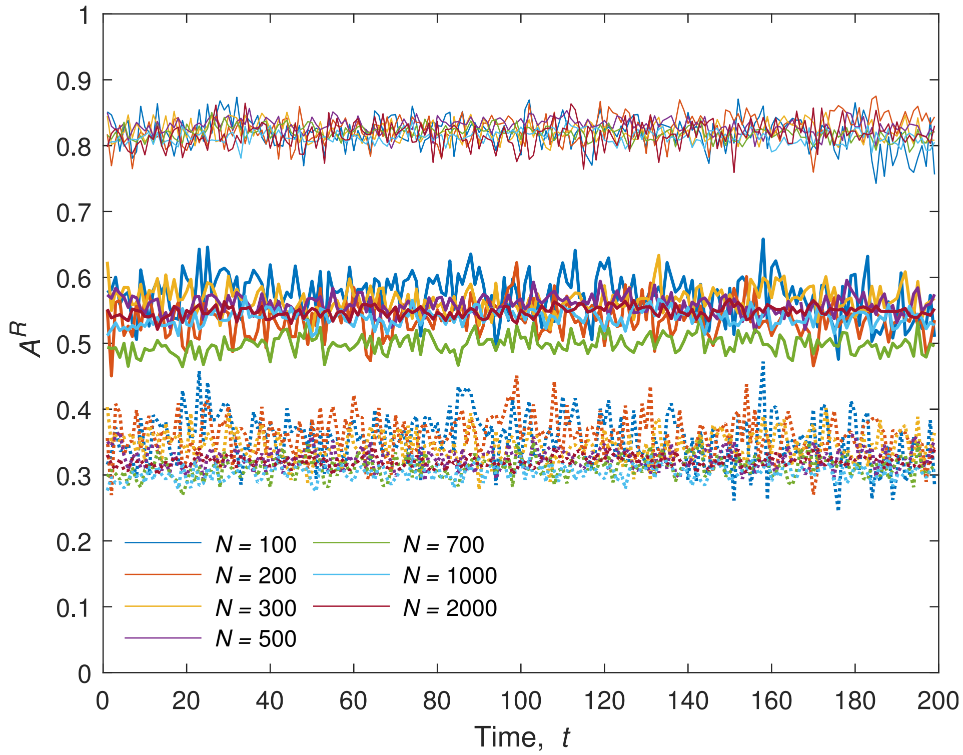

Supplementary Figure 8. The time evolution of A^R dependent on the number of agents $N = 100, 200, 300, 500, 700, 1000, 2000$ for $R = R_0$ (thin solid lines), $1.6R_0$ (thick solid lines), and $2.0R_0$ (dashed lines) at high noise level ($\eta_0 = \frac{11\pi}{6}$). Here, R_0 is 34.7 pixels ($N = 100$), 24.5 (200), 20 (300), 15.5 (500), 13 (23), 11 (1000), and 7.75 (2000). On the top of Fig. 6 (main text) showing the existence of the universal feature of A^R landscape in the normalized plane of $(\frac{R}{R_0}, \frac{\gamma}{\gamma_0})$ space for $100 \leq N \leq 1000$, this figure demonstrates that, once R is normalized by the factor of R_0 dependent on N, the alignment score A^R is almost free from the choice of N.

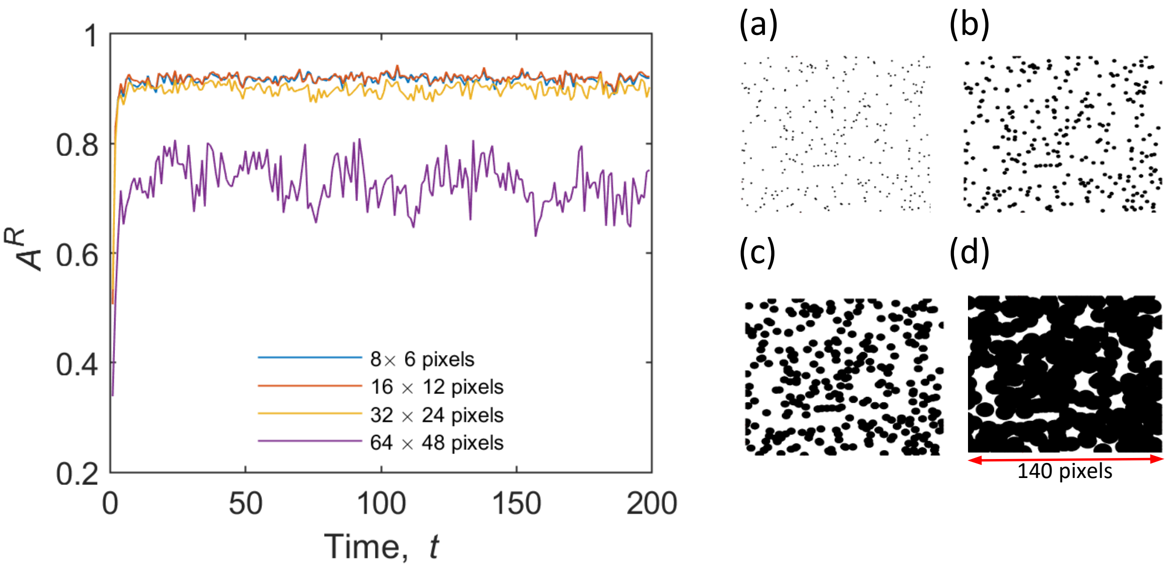

Supplementary Figure 9. Time developments of the alignment score A^R for different particle sizes at noise $\eta_0 = \pi/2$ with $N = 300$, $R_0 = 20$ pixels and the PIV grid size γ =64 pixels. A^R appears consistent as long as the particle size is small enough compared to γ. The alignment score A^R starts to decrease drastically as the particle size approaches the grid size γ. A representative snapshot of particles with different sizes, where the sizes are (a) 8×6, (b) 16×12, (c) 32×24, and (d) 64×48 pixels. In this simulation, we kept the original Vicsek model so that we simply added ovals of the finite sizes on the positions of Vicsek particles without a hard/soft collision potential. In (a)-(d), the positions of the particles are the same but their sizes are different.
